# Protometabolic functions of pyridoxal: A link between early amino acid synthesis and enzyme evolution

**DOI:** 10.1111/febs.70056

**Published:** 2025-03-07

**Authors:** Mariarita Bertoldi, Gianluca Molla

**Affiliations:** ^1^ Dipartimento di Neuroscienze, Biomedicina e Movimento Università degli Studi di Verona Italy; ^2^ The Protein Factory 2.0, Dipartimento di Biotecnologie e Scienze della Vita Università degli Studi dell'Insubria Varese Italy

**Keywords:** amino acid synthesis, enzyme evolution, pyridoxal, pyridoxamine

## Abstract

In the framework of studies on protometabolism, Schlikker *et al*. characterized the conversion of pyridoxal to pyridoxamine under conditions mimicking the ones likely existing at the origin of metabolism. These conditions triggered nitrogen incorporation into amino acids in solution before the origins of enzymes. The suggested role for pyridoxal highlights its pivotal function in the transition from inorganic ammonia‐dependent amino acid synthesis to organic reactions in aqueous solution and supports the “metabolism first” theory for biological evolution. Insights from the early evolution of natural enzymes can inspire the development of novel biocatalysts for biotechnological applications based on the catalytic versatility of pyridoxal.

## Graphical abstract

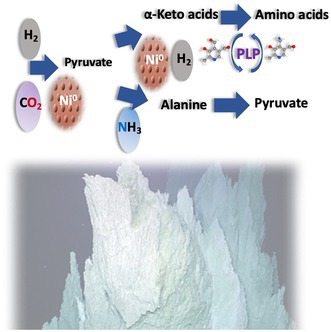

**Abbreviations**
PL: pyridoxal
PLP: pyridoxal 5′‐phosphate
PM: pyridoxamine
PMP: pyridoxamine 5′‐phosphate

## The catalytic versatility of pyridoxal (PL) and pyridoxal 5′‐phosphate (PLP)

**Fig. 1:**
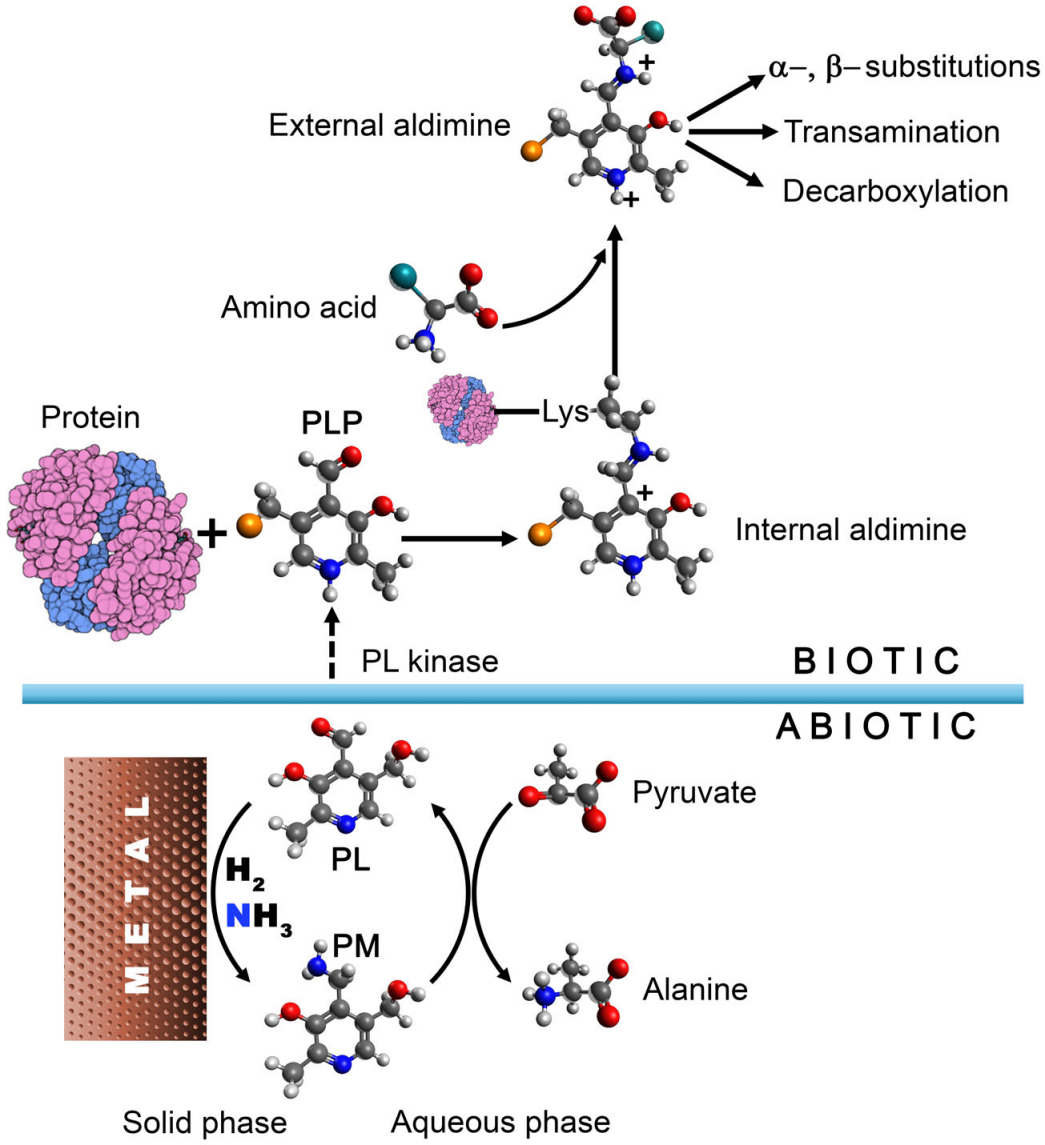
The evolution of versatile pyridoxal 5′‐phosphate (PLP) enzymes. In the abiotic world, pyridoxal (PL) can be aminated to pyridoxamine (PM) through a metal‐catalyzed reaction on solid‐phase catalyst surface. PM serves as an amino donor to oxoacids for the conversion into amino acids in solution. In the biotic world, through a phosphorylation reaction mediated by a kinase, PL converts into pyridoxal 5′‐phosphate (PLP), which is incorporated into PLP‐enzymes through a Schiff base linkage (internal aldimine). The entrance of an amino acid at the active site of PLP‐enzymes leads to the formation of the external aldimine intermediate, which is prone to catalyzing a variety of reactions whose specificity depends on the protein moiety. The coenzyme's structures are reported as ball‐and‐stick models colored by CPK.

PL is the aldehydic form of pyridoxine, or vitamin B_6_, which derives from pyridine and is used as a chemical skeleton for the synthesis of other B_6_ vitamers, such as pyridoxamine (PM) and their 5′‐esterified phosphorylated derivatives with biological activity: PLP and pyridoxamine 5′‐phosphate (PMP). PLP is ubiquitous in nature and is required as an essential coenzyme for reactions involving amino acid metabolism and glycogen catabolism, comprising at least 4% of classified enzyme activities [1]. The biological *de novo* synthesis of pyridoxine occurs in plants and microorganisms. Animals absorb B_6_ vitamers from their diet and convert them into oxidized and phosphorylated active PLP through a salvage pathway requiring the ubiquitously expressed PL kinase and pyridoxine/pyridoxamine 5′‐phosphate oxidase [2]. At the enzyme active sites, PLP is bound via a covalent Schiff base linkage (or internal aldimine) to the amine side chain of a lysine residue; this holoenzyme complex represents the resting and fully functional catalytic form. If an amino acid or amine‐containing substrate enters the active site, a nucleophilic attack takes place, generating a Schiff base with the amine group of the substrate (the external aldimine) via a transaldimination reaction through an intermediate *gem*‐diamine species. The protein moiety then directs the fate of the external aldimine by orienting it so that a specific reaction is promoted, while alternative paracatalytic activities, due to the intrinsic PLP catalytic versatility, are suppressed (reviewed in [3]) (Fig. 1). Indeed, the primary driver of the association between enzymes and cofactor was the ability of the protein moiety to modulate the reactivity of the cofactor and to increase its stability, as recently proposed by [4]. In PLP‐dependent enzymes, the role of the protein moiety is unrelated to the cofactor's inherent chemical capability, but it is confined to the relevant enhancement of the reaction efficiency (from 10^10^‐ to 10^18^‐fold) [5] and the suppression of the unwanted side reactions.

Schlikker *et al*. [6] contribute to advancing the understanding of how amination reactions, that is, incorporation of nitrogen into biological chemistry and organic reactions, evolved from the solid‐state metal‐catalyzed surface to PL‐catalyzed transamination in an aqueous phase, allowing the employment of PM for amino acid synthesis and the subsequent evolution of PLP‐enzymes in cells. In detail, the authors provide evidence that (i) H_2_ and ammonia over Ni^0^ favor the conversion of PL to PM, and (ii) PL significantly impacts the reductive amination of pyruvate into alanine under conditions of active serpentinization [7]. Through these findings, the study takes a step forward in understanding the transition of protometabolism from solid surfaces to an aqueous *milieu*, paving the way toward a more biological environment similar to that present in cells.

## The framework of theories of the development of modern enzymes

About 50% of enzymes rely on cofactors for catalysis [8]. It is plausible that this is also true for the most ancient enzymes, as inferred from the reconstructions of the ancestral genomes [9]. The extreme evolutionary conservation of current protein cofactors strongly suggests that they are very ancient [10] and that they likely originated from inorganic reactions in a suitable geochemical environment in which life began.

Many cofactors display significant levels of catalytic activity across a diverse range of chemical reactions. This also applies to PLP, which is inherently able to catalyze a plethora of reactions even in the absence of the protein moiety: transaminations, α‐ and β‐substitutions, and decarboxylation of amino acids can occur in the presence of PLP and metal ions [11, 12]. Thus, the described amination of PL to PM (the precursors of PLP to PMP) [6] and the use of PM as a source of aminic groups for oxoacids represent a pathway to incorporate nitrogen into organic compounds in an abiotic environment.

Both polypeptides and cofactors were likely available already at a prebiotic stage [13]. Therefore, it is conceivable that the presence of primordial polypeptides at some early evolutionary stage promoted the association of ancestral cofactors to the protein moiety to enhance their catalytic power, stability, and effective local concentration.

Hence, the recruitment of catalytic cofactor PL by polypeptides, as elegantly proposed by Schlikker *et al*. [6], provides a simple and straightforward mechanism for the *de novo* emergence of many of the primordial enzymes, providing valuable hints for the design of novel biocatalysis for biotechnological applications (see below).

## Molecular evolution at hydrothermal vents (a time‐traveler location) and the theory of “metabolism first”

Conditions at serpentinizing hydrothermal vents are peculiar but have fewer extremes in comparison to black smokers, such as moderate temperature (< 200 °C, much lower than the typical black smokers' one ~400 °C), pressure typically around 200–400 bar in deep‐sea environments, alkaline pH (9–12) due to hydroxide production from serpentinization, and low oxygen concentration. These properties, together with the presence of dissolved H₂ (deriving from olivine‐water reactions), favor prebiotic chemistry and promote the formation of abiotic methane, produced from the CO_2_ reduction and small organic molecules (such as formate and acetate). The work by Schlikker *et al*. [6] shows that such hydrothermal vents could represent a key environment for prebiotic chemistry simulating early Earth conditions. The authors' findings support the “metabolism first” theory, which opposes the “genetics first” hypothesis, suggesting that carbon and nitrogen entered prebiotic chemistry through reaction routes that strongly resemble the modern enzymatic pathways. According to this theory, inorganic surfaces and cofactors present in such environments served as catalysts at the origin of metabolism [14], and, consequently, the first organisms were autotrophs able to obtain their carbon from CO_2_.

High temperatures observed at serpentinizing hydrothermal vents could have accelerated slow reactions, such as the amination reactions discussed in the paper, which were promoted by primordial cofactors such as PL. These conditions likely shortened the time required for primordial chemistry to establish itself. Subsequently, the slow but steady cooling of the hot primordial environment resulted in a tremendous evolutionary force that acted to increase the catalytic power of ancestral enzymes. This is because, with the decrease of the temperature, the need for catalysts that reduce the activation energy barrier of reactions allowed us to maintain suitable reaction rates even at the slowly appearing moderate temperatures. This process also included the holoenzymes binding primitive abiotic cofactors (*e.g*., PL). Thus, serpentinizing conditions, replicating the early evolution condition of life on Earth, provide an appropriate setting for exploring the vast range of chemical possibilities and for the rapid evolution of primordial enzymes [7].

## New insights from ancestral biocatalysts

Highlighting the connection between primordial catalytic mechanisms and the engineering of biocatalysts is an intriguing area of research. The insights on the origin of primordial enzymes not only expand our understanding of molecular evolution but also provide valuable guidelines for practical biotechnological applications aimed at environmental sustainability, such as CO_2_ fixation or enzymatic valorization of waste feedstocks.

Indeed, the generation of efficient enzymatic activity not yet present in nature by protein engineering of existing enzymes or *de novo* protein design is still extremely challenging [15], particularly for cofactor‐dependent enzymes. However, original primordial holoenzymes must have necessarily arisen *de novo*, likely from the interaction between noncatalytic protein scaffolds and abiotic primordial cofactors (such as PL), whose abiotic synthesis from glycolaldehyde and ammonia or through alternative routes is possible in the presence of high temperatures [16]. Early enzymes were likely generalists in both their catalyzed reactions and their substrate ranges. This catalytic promiscuity granted early biocatalysis wide versatility that was crucial in the framework of primordial metabolic networks. These biocatalysts have been later subjected to natural molecular selection and evolution, resulting in present days efficient enzymes. Understanding the kinetic properties, catalytic mechanisms, and evolution mechanisms of ancestral enzymes can provide researchers with valuable information to redesign modern biocatalysts with increased or changed substrate scope and/or improved activity and stability, rendering them suitable for biotechnological application, especially in the field of biocatalysis for feedstock valorization. In this regard, the application of enzymes as catalysts for the synthesis of different products has proven to be more efficient and a much greener approach in contrast to its inorganic counterparts [17]. Specifically, from an applicative point of view, the information provided by Schlikker *et al*. [6] will be useful for the *in vitro* evolution of PLP‐dependent enzymes, which are currently exploited in several biotechnological applications, mainly as biocatalysts in industrial biosynthetic processes [18].

## Conflict of interest

The authors declare no conflict of interest.

## Author contributions

MB and GM conceptualized and wrote the manuscript.
